# Free but declining: the paradox of contraceptive uptake in Burkina Faso

**DOI:** 10.3389/frph.2026.1819570

**Published:** 2026-08-05

**Authors:** Marie-Jeanne Offosse, Tozé Namalela, Obasanjo Bolarinwa, Awa Yanogo, Yeri Traoré, Aduragbemi Banke-Thomas

**Affiliations:** 1 Health Systems Insight (formely ThinkWell Institute), Ouagadougou, Burkina Faso; 2 Health Systems Insight (formely ThinkWell Institute), Maputo, Mozambique; 3 Department of Global Healthcare Management, York St John University, London, United Kingdom; 4 Family Health Department, Ministry of Health and Public Hygiene, Ouagadougou, Burkina Faso; 5 Maternal, Adolescent, Reproductive and Child Health Centre, London School of Hygiene and Tropical Medicine, London, United Kingdom

**Keywords:** Burkina Faso, family planning service utilization, modern contraceptive, stock-outs, geographic inequity, adolescent utilization

## Abstract

**Introduction:**

In 2020, Burkina Faso expanded its national user fee exemption policy to include family planning services, with the objective of reducing financial barriers and increasing uptake of modern contraceptive methods. This study assesses the national and subnational trends in modern contraceptive uptake before and after the user fee exemption policy amongst women of reproductive age (WRA) in Burkina Faso.

**Methods:**

To achieve this objective, we collected routine health facility and demographic data spanning the pre-intervention period (2016-2018) and the post-intervention period (2021-2024). Mixed-effects linear models examined district-level variations in modern contraceptive uptake, while descriptive analyses explored group differences and temporal trends in contraceptive uptake among WRA between 2021 and 2024.

**Results:**

At the national level, overall contraceptive uptake (all methods) declined by 3.26 percentage points between the pre- and post-policy periods (*p* < 0.01). The largest reductions were observed in Boucle du Mouhoun (−9.49 percentage points), Sahel (−8.6 percentage points), and Nord (−6.75 percentage points) regions (all *p* < 0.01). In the post-implementation period, modern contraceptive uptake among WRA declined by 3.6 percentage points nationally, with downward trend observed across all age groups but more pronounced among adult women. Modern contraceptive uptake among adult women declined by 4.5 percentage points, from 24.1% in 2021 to 19.6% in 2024. In contrast, adolescents experienced a smaller decline of 0.7 percentage points, from 16.3% to 15.6% over the same period (*p* < 0.01). Amongst women who sought care in health facilities in stable areas, modern contraceptive uptake declined by 3.4 percentage while security-challenged areas experienced a slightly higher decline of 3.6 percentage points, from 24.0% to 20.6% (*p* < 0.01). Similarly, facilities in urban areas experienced a lower decline (−1.1 percentage points) in modern contraceptive uptake compared to those in rural area (−5.2 percentage points (*p* < 0.01). Nearly one-third of health facilities reported stock-outs exceeding three months for some methods.

**Conclusion:**

The removal of user fees for family planning services in Burkina Faso was insufficient to reverse declining modern contraceptive uptake with adult women and those living in rural and conflict-affected areas benefiting least. These findings highlight the need for complementary non-financial strategies to improve potential gains of the user fee exemption policy.

## Background

In 2023, nearly 70% of the estimated 260,000 maternal deaths and about 50% of the two million stillbirths that occurred globally were in sub-Saharan Africa (SSA) (1, 2). These deaths have been partly attributed to limited access to and utilisation of family planning commodities (3). Family planning has been proven to deliver a wide range of health and social benefits, most notably substantial reductions in maternal and perinatal mortality (4, 5). Expanding access to effective family planning has the potential to avert over 40% of maternal deaths and nearly 10% of childhood deaths (6, 7). This substantial preventive potential underpins the significant investments made over the past five decades by governments, non-governmental organisations, multilateral agencies, and civil society to strengthen access to family planning services across the region (8, 9).

On average, use of modern contraceptives, such as male and female condoms, oral contraceptive pills (OCPs), intrauterine devices (IUDs), emergency contraception, injectables, sterilisation, and implants in SSA increased from just over 8% in the 1980s to about 22% by 2010 (10). The rate increased to 56% by 2022, however, this is lower than in other regions of the world, despite extensive investment by international donors and efforts by national governments over the preceding two decades (11, 12). In addition to demand side issues such as socio-cultural and religious barriers, misinformation and fear, and low education and poverty, supply side issues relating to limited access and availability have been attributed to this relatively low rate of modern contraceptive use in SSA (13, 14).

In response to these issues, many countries in SSA adopted health financing reforms aimed at reducing direct out-of-pocket costs for reproductive health services (15). User fee exemption policies, in particular, have been widely implemented as a strategy to improve access, stimulate demand, and promote equity in the utilisation of essential services, including family planning (13). Within this policy landscape, Burkina Faso expanded its national user fee exemption policy, Gratuité, in 2020 to include family planning services, after a six-month pilot in 2019 conducted in two regions. This extension to cover family planning services was informed by the improvement of maternal newborn and child health results through the Gratuité scheme, as well as the success of national free family planning weeks (*Semaines Nationales de la Planification Familiale*) held annually (16). The reform was explicitly designed to remove financial barriers at the point of care, covering 100% of the cost relating to consultation, counselling, tests, examinations, modern family planning commodities and consumables for women of reproductive age (WRA, comprising women aged 15–49 years), particularly targeting vulnerable women, including adolescents and those in humanitarian-affected regions (17–20), who are known to have have higher likelihood of unmet need for family planning (21, 22). However, in reality, while consultation and counselling was free, the reform leveraged existing supply platforms of family planning commodities to ensure supply (16).

To the best of our knowledge, there is only one previous study that assessed the effect of the Gratuité policy on use of modern contraception. This study was conducted in select districts of Burkina Faso and compared use amongst WRA during the pilot months and a year after national rollout comparing four pilot and four non-pilot districts (23). However, knowledge gaps remain regarding the effect of the policy across the entire country and its the long term effect on family planning uptake beyond the immediate timeline after its implementation compared to before the pilot. Also, there is still a need to understand uptake since the policy was implemented within the context of its reliance on existing structures to ensure supply of family planning commodities. Demand without supply ultimately mean sustainability of any meaningful health system gains, including reductions in unintended pregnancies and maternal morbidity and mortality, will be limited. To address these gaps, our objective in this study was to undertake a national-level evaluation of modern contraceptive uptake two years before the pilot and four years after the national roll out of the user fee exemption policy for free family planning services in Burkina Faso. The findings from the study will provide robust national evidence to inform policy and advocacy efforts, including ongoing initiatives by civil society organisations within the Ouagadougou Partnership to strengthen free family planning provision and promote more equitable access to services across the country and generate lessons for similar settings.

## Methods

### Study design and setting

This study is a trend analysis of repeated cross-sectional health facility-based surveys from 2016 to 2024, allowing comparison of annual modern contraceptive uptake between the pre-implementation period (2016–2018) and the post-implementation period (2021–2024). Complete annual uptake data for years 2019 and 2020 were not available for different reasons. For 2019, this was the pilot year and there was a documented data gap for three months of the year due to a national strike (16, 24); while year 2020 coincided with the national rollout of the free family planning services which did not actually commence until July of that year (16).

Burkina Faso is a landlocked Sahelian country in West Africa and part of SSA. It covers approximately 274,200 km^2^ and shares borders with Mali, Niger, Benin, Togo, Ghana, and Côte d'Ivoire. In 2019, Burkina Faso had an estimated population of just over 20 million of which women accounted for approximately 52% (25). The annual population growth rate was approximately 3%, and by 2025 the population was projected to reach over 24 million, with over 70% residing in rural areas. WRA represent approximately 24% of the total population. The total fertility rate is estimated at 4 births per woman, with adolescent fertility contributing substantially to overall fertility levels. The country's gross domestic product per capita is US$857, lower than the average across countries in SSA of US$1,501 and US$10,918 globally (26). As of 2018, an estimated 41% of the Burkinabè population lives below the national poverty line of US$1.90 daily (25).

The health system in Burkina Faso is organised into three levels: district, regional, and national. Family planning services are primarily delivered at the district level through a network of primary healthcare facilities, comprising 2,266 Health and Social Promotion Centres (Centres de Santé et de Promotion Sociale, CSPS) and 144 Medical Centres (Centres Médicaux, CM). CSPS facilities serve as the first point of contact within the primary healthcare system and provide a minimum package of services, including family planning (27). The responsibility for contraceptive procurement and supply chain is shared by the national government and donors. While donor dependency was described as high, the Burkinabè government was commended for allocating 37% and spending 25% of the funds needed to purchase contraceptives in 2020 (28). The Central Purchase of Generic Essential Drugs and Medical Consumables known locally as Centrale d'Achat des Médicaments Essentiels Génériques et des consommables médicaux (CAMEG) is the central purchasing, storage, and distribution center for pharmaceutical products, medical commodities, supplies, and equipment serving both public and private health faciltiies operating in Burkina Faso that provide public health services including provision of contraceptives (29). On the demand side, most modern contraceptive users (81%) in Burkina Faso obtain their method from a public health facility (30). In terms of strategy, the country set a national target of 40% uptake of modern family planning (20), building on an estimate of 30% reported in the Performance Monitoring and Accountability 2020 Round 6 survey in Burkina Faso which was conducted between late 2018 and early 2019 (31).

### Data sources and assembly

We sourced data from routinely collected health and demographic data from Burkina Faso to be able to capture both numerator and denominator required for service utilisation analysis, respectively.

For the numerator, we sourced data from the Ministry of Health's District Health Information System 2 (DHIS2). DHIS2 is an open source, health management data platform, used as part of the national health information system in many countries in SSA including Burkina Faso. DHIS2 typically includes facility-level records on service delivery which can be used for data management and analysis, health program monitoring and evaluation, and service availability mapping, for logistics management (32). From this platform, family planning service data, including numbers of family planning clients, new clients, method type, and contraceptive stock-outs, were extracted.

Population denominator for WRA was based on catchment populations reported in the Ministry of Health's Master Facility List 2025, which also reports other facility-level data, including region, district, commune, urban–rural classification, catchment area, distance to facilities from serviced villages within the catchment area, and population counts disaggregated by sex. Population denominators for the study period (2016–2024) were reconstructed retrospectively using province-level demographic projections from the 4th (2006) and 5th (2019) General Population and Housing Censuses (RGPH) published by the National Institute of Statistics and Demography (INSD) in Burkina Faso. The 2007–2020 projections were based on the 4th RGPH (33), while the 2020–2035 projections were derived from the 5th RGPH (34). Both projection series incorporated assumptions regarding fertility, mortality, and migration and provided annual estimates disaggregated by sex, age group, region, and province (33, 34).

Data from both numerator and denominator datasets were matched. Health facilities included in the final analysis were those successfully matched across population coverage and family planning datasets. Some facilities were excluded at this point due to inconsistencies in naming across data sources, which prevented reliable linkage. Following these exclusions, the matched dataset included data from 2,089 health facilities across all 13 regions and 70 districts. For our analysis, each health facility was mapped to its corresponding province, since province represents the lowest level of disaggregation available in the INSD projections. Province-specific proportions of WRA and annual population growth rates were calculated and applied to facility catchment area population counts to estimate annual WRA denominators for each health facility.

### Study variables

The primary outcome indicator captured overall family planning uptake and included the use of any contraceptive method and the use of modern contraceptive methods. Contraceptive uptake was defined as the use of modern (including condoms, OCPs, IUDs, emergency contraception, injectables, implants, and sterilisation) or traditional (lactational amenorrhoea method through exclusive breastfeeding and periodic abstinence based on fertility awareness, such as calendar-based or rosary-based methods) methods, as classified within the DHIS2. We further disaggregated the modern contraceptive methods by method category (short-acting or long-acting methods) and the specific modern contraceptive method, including condoms (female condom and male condom), self-administered pills (progestin-only pills and combined oral pills, and emergency contraception, levonorgestrel), a provider-administered implants (levoplant, Jadelle, and Implanon), a self-administered injectable method (Sayanapress), a provider administered injectable (Depo-Provera), provider administered IUD, and sterilisation (tubal ligation and vasectomy).

From DHIS2 (32), we extracted data for four key variables: age group of WRA [categorised as adolescents (15–19 years) and adults (20–49 years)], location of facility (urban or rural), security context of facility (stable areas or areas affected by security challenges), and contraceptive stock-out (unavailability of a specific contraceptive commodity), measured as the percentage of health facilities experiencing stock-outs lasting more than three consecutive months—a duration agreed upon and utilised jointly by the world's major health and development bodies to ensure consistency in research and supply chain monitoring, and which has been used in previous similar research (35, 36).

### Statistical analysis

Descriptive statistics, using frequencies and percentages were used to describe characteristics of included health faciltiies. Subsequently, mean uptake for any family planning was calculated nationally and per region, comparing pre-policy (2016–2018) and post-policy (2021–2024), and variance estimated. Interrupted time series analysis using segmented regression was employed to assess changes in family planning uptake trends associated with the introduction of free family planning policy (37, 38). The model estimated baseline level, pre-intervention trend, immediate level change following the intervention, and change in trend post-intervention, using the Wilcoxon paired test. Covariates for urban/rural residence and area stability were included. Statistical significance was assessed using t-tests with 95% confidence intervals.

Analysis of modern contraceptive uptake focused on the post-implementation period from 2021 to 2024 since routine health information systems in Burkina Faso only began reporting family planning uptake by method type from 2021. For this analysis, descriptive analyses and mixed-effects linear models were used to examine modern family planning uptake during the post-implementation period and to assess factors associated with trends in family planning upkate while accounting for clustering at the district level (39). Analysis was stratified by age group, region, contraceptive method type, location of facility, and security status of facility district.

A descriptive analysis of routine health facility data was conducted for the post-implementation period to estimate the proportion of CSPS experiencing contraceptive stock-outs for three months. For each year and contraceptive method, the proportion of CSPS reporting prolonged stock-outs was calculated by dividing the number of facilities with stock-outs exceeding three months by the total number of reporting CSPS and expressing the result as a percentage. Estimates were produced at the national level and stratified by security status of facility district (security-challenged vs. stable areas), based on official administrative classifications. Commodities introduced during the study period were analysed only for years in which data were available.

Statistical and spatial analyses were conducted in RStudio version 4.4.2 and QGIS version 3.40.11, respectively.

## Results

### Characteristics of health facilities

Of the 2089 CSPS included in the analysis, 1542 (73.8%) were located in rural areas and the remaining 547 (26.2%) were in urban areas. In addition, 1281 (38.7%) of the facilities were situated in stable areas while 808 (38.7%) were in districts affected by security challenges.

### Trends in family planning uptake (all methods) before and after the introduction of free services

At the national level, family planning uptake, considering all methods, declined by 3.26 percentage points, from 24.3% in the pre-policy period to 21.3% in the post-policy period (*p* < 0.01). By region, there were significantly larger reductions recorded in Boucle du Mouhoun (−9.49 percentage points) (*p* < 0.01), Sahel (−8.6 percentage points) (*p* < 0.01), and Nord (−6.75 percentage points) regions (*p* < 0.01) (Table 1).

**Table 1:** Mean contraception uptake for WRA pre- and post-free family planning policy.

Region	Pre-policy (2016–2018)	Post-policy (2021–2024)	Variance	95% CI	*p*
Boucle du Mouhoun	28.1%	18.58%	−9.48%	[−11.3%: −7.7%]	<0.01
Cascades	23.0%	19.29%	−3.75%	[−4.9%: 0.6%]	0.1241
Centre	18.9%	17.05%	−1.90%	[−1.1%: 4.8%]	0.2162
Centre Est	18.7%	18.90%	0.20%	[−0.97%: 1.97%]	0.5005
Centre Nord	27.9%	30.69%	2.82%	[−5.5%: 0.94%]	0.1657
Centre Ouest	23.5%	18.39%	−5.08%	[−5.6%: −1.99%]	<0.01
Centre Sud	32.2%	27.90%	−4.33%	[−5.8%: −0.3%]	0.0341
Est	20.6%	18.72%	−1.87%	[−2.5%: 2.4%]	0.9824
Hauts Bassins	23.3%	20.57%	−2.73%	[−6.1%: −2.1%]	0.0001
Nord	29.9%	23.15%	−6.75%	[−10.1%: −6.5%]	<0.01
Plateau Central	25.3%	25.19%	−0.12%	[−2.2%: 2.4%]	0.9256
Sahel	27.1%	18.53%	−8.60%	[−15.2%: −5.8%]	<0.01
Sud Ouest	28.9%	26.68%	−2.25%	[−4.2%: 0.8%]	0.1757
National	24.3%	21.03%	−3.26%	[−4.3%: −2.98%]	<0.01

### Trends in modern family planning uptake by population and facility of uptake characteristics following the introduction of the free family planning policy

Between 2021 and 2024, modern contraceptive uptake among WRA in Burkina Faso declined by 3.6 percentage points nationally, reflecting a gradual annual decrease from 22.3% in 2021 to 18.7% in 2024. This downward trend was observed across both age groups (adolescents and adult women) but was more pronounced among adult women. Modern contraceptive uptake among adult women declined by 4.5 percentage points, from 24.1% in 2021 to 19.6% in 2024 (*p* < 0.01). However, adolescents experienced a smaller decline of 0.7 percentage points, from 16.3% to 15.6% over the same period (*p* < 0.01) (Table 2).

**Table 2:** Proportion of women who received modern contraceptive methods in Burkina Faso post policy implementation, by client and facility characteristics.

Modern contraceptive uptake	2021	2022	2023	2024	Variance	Relative variation	*p*
National	22.3%	19.3%	19.0%	18.7%	−3.60%	−16.14%	<0.01
Age groups							
Adolescents (15–19 years)	16.3%	15.2%	15.7%	15.6%	−0.70%	−4.29%	<0.01
Adult women (20–49 years)	24.1%	20.5%	20.0%	19.6%	−4.50%	−18.67%	<0.01
Geographic location of facility							
Urban	19.8%	18.2%	18.6%	18.7%	−1.10%	−5.56%	<0.01
Rural	23.9%	20.0%	19.3%	18.7%	−5.20%	−21.76%	<0.01
Security status of facility district							
Security challenged	24.0%	19.9%	19.5%	20.4%	−3.60%	−15.00%	<0.01
Stable area	21.2%	19.0%	18.7%	17.8%	−3.40%	−16.04%	<0.01

Declining patterns were also observed in disaggregated analysis by security contexts of facilities of uptake, although the magnitude of change differed. Amongst women who sought care in facilities in stable areas, modern contraceptive uptake significantly declined by 3.4 percentage points, from 21.2% in 2021 to 17.8% in 2024, while those in security-challenged areas experienced a slightly higher significant decline of 3.6 percentage points, from 24.0% to 20.4% (*p* < 0.01). Similarly, the declining pattern differed by geograhic location. WRA in facilities located in urban areas experienced a significant lower decline (−1.1 percentage points) in modern contraceptive uptake compared to those in rural area (−5.2 percentage points) (Table 2).

**Figure 1:**
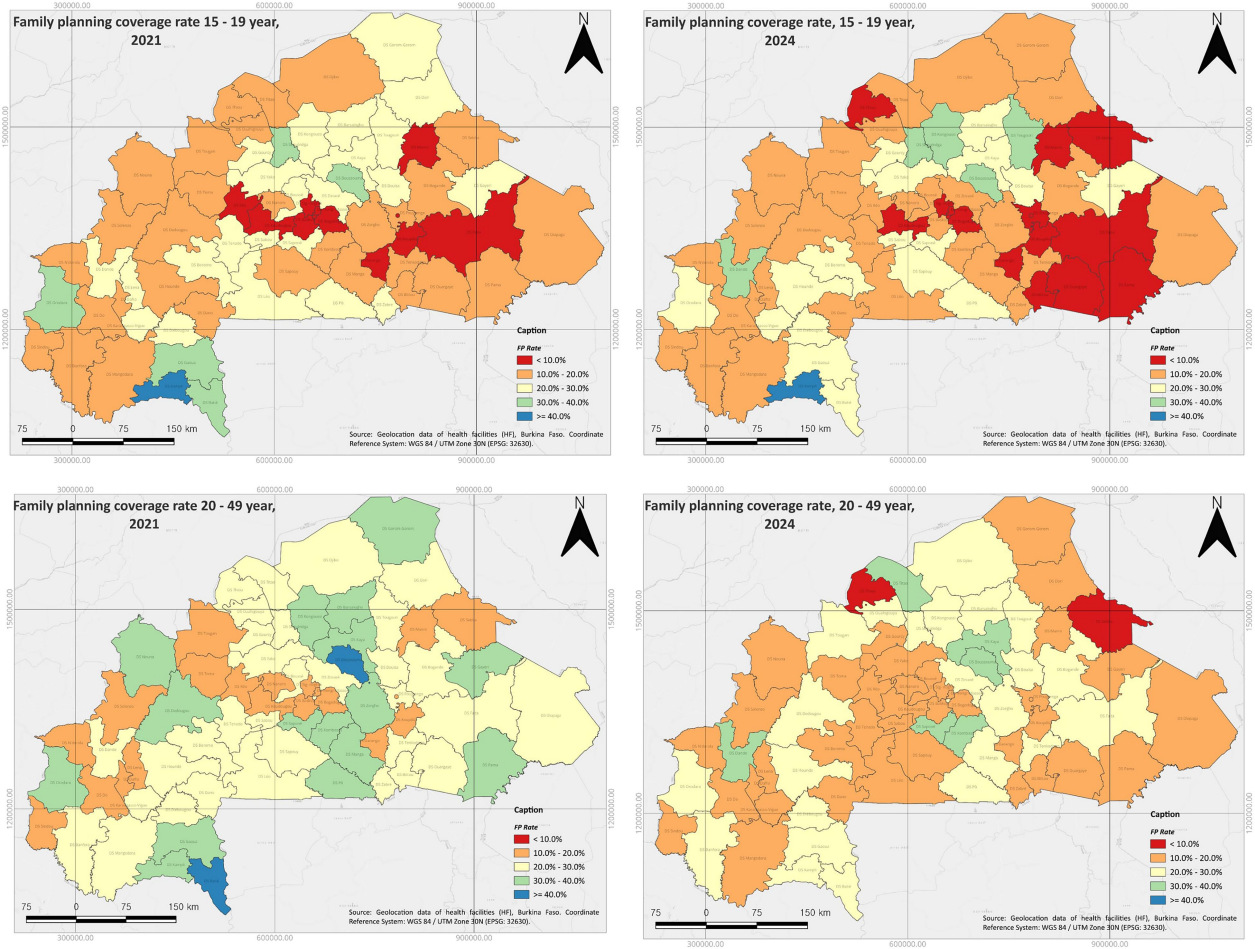
Modern family planning methods uptake in 2021 and 2024 among adolescents (15–19 years) and adults (20–49 years), by district.

There was a general decline in family planning uptake across districts, specifically in Thiou, Sebba, Bittou, Ouargaye, Pama, Orodara, Gaoua and Batié districts, alongside substantial district heterogeneity, particularly by age group and security status of the uptake facility. Furthermore, there was persistent low uptake of modern family planning compared to the national target (>40%) (Figure 1).

A gradual shift towards long-acting methods was observed among modern family planning clients over the study period. The proportion of WRA who received short-acting methods declined by 3.1 percentage points, from 57.3% in 2021 to 54.2% in 2024, while long-acting methods uptake increased by a corresponding magnitude, from 42.7% to 45.8%. Despite this shift, short-acting methods remained the predominant form of modern contraceptive. Subgroup analysis indicated that this transition towards long-acting methods was driven primarily by rural areas, while method uptake patterns remained relatively stable in urban areas. In contrast, the shift was observed consistently across both stable and security-challenged areas (Table 3).

**Table 3:** Short acting vs. long-acting modern contraceptive methods uptake by facility characteristics (2021–2024).

Method type		Short-acting				Long-acting				*p* value
Year		2021	2022	2023	2024	2021	2022	2023	2024	
National		57.30%	54.50%	54.70%	54.20%	42.70%	45.50%	45.30%	45.80%	<0.01
Geographic location	Rural	58.10%	55.20%	54.90%	53.50%	41.90%	44.80%	45.10%	46.50%	<0.01
	Urban	55.80%	53.30%	54.40%	55.30%	44.20%	46.70%	45.60%	44.70%	0.5974
Security status	Security challenged	59.60%	55.90%	55.90%	54.70%	40.40%	44.10%	44.10%	45.30%	<0.01
	Stable	55.70%	53.70%	54.00%	53.90%	44.30%	46.30%	46.00%	46.10%	<0.01

Analysis of specific modern family planning method uptake revealed heterogeneous trends during the post implementation phase. Overall, uptake of male condoms statistically significantly declined by 2.5 percentage points (*p* < 0.01), alongside a statistically significant downward trend in OCP uptake, including progestin-only pills (−0.3 percentage points) and combined OCP (−0.6 percentage points). On the other hand, there was a statistically significant upward trend in the uptake of implant-based methods such as Implanon (+3.0 percentage points), and Levoplant (+0.9 percentage points), as well as in IUD uptake (0.7 percentage points). Separately, uptake of Sayana Press, a self-administered implant, increased significantly among WRA, rising by 5.2 percentage points from 19.2% in 2021 to 24.5% in 2024. In contrast, Depo-Provera, a provider-administered injectable, significantly declined by 7.9 percentage points, from 35.3% to 27.4% over the same period. All observed differences were statistically significant (*p* < 0.01) (Table 4).

**Table 4:** Preferred modern contraceptive methods among family planning users, by age group (2021–2024).

Method type	Methods	2021	2022	2023	2024	2021–2024 variation	*p*
National							
Short acting contraceptive methods	Female condom	0.1%	0.1%	0.1%	0.1%	0.00%	0.10
	Male condom	6.0%	2.6%	2.8%	3.5%	−2.50%	<0.001
	Progestin-only pills	2.0%	1.9%	2.0%	1.7%	−0.30%	<0.001
	Combined OCPs	9.5%	9.9%	9.1%	8.9%	−0.60%	<0.001
	Norlevo	0.0%	0.0%	0.00%	0.2%	0.20%	<0.001
	Sayanapress	19.2%	20.7%	22.9%	24.5%	5.30%	<0.001
	Depoprovéra	35.3%	33.2%	30.3%	27.4%	−7.90%	<0.001
Long acting contraceptive methods	Levoplant	0.0%	0.0%	0.00%	0.9%	0.90%	<0.001
	Jadelle	22.8%	25.0%	26.3%	24.3%	1.50%	0.193
	Implanon	2.9%	4.2%	3.9%	5.9%	3.00%	<0.001
	IUD	2.1%	2.3%	2.5%	2.7%	0.60%	<0.001
	Vasectomy	0.00%	0.00%	0.00%	0.00%	0.00%	0.531
	Tubal ligation	0.01%	0.00%	0.01%	0.00%	−0.01%	0.7036
Adolescents							
Short acting contraceptive methods	Female condom	0.1%	0.1%	0.2%	0.1%	0.00%	0.075
	Male condom	6.7%	2.6%	2.6%	3.2%	−3.50%	<0.001
	Progestin-only pills	1.4%	1.3%	1.3%	1.1%	−0.30%	<0.001
	Combined OCPs	6.5%	6.5%	6.1%	6.5%	0.00%	<0.001
	Norlevo	0.0%	0.0%	0.0%	0.1%	0.10%	<0.001
	Sayanapress	18.7%	19.7%	22.0%	22.9%	4.20%	<0.001
	Depoprovéra	27.9%	26.4%	23.6%	21.1%	−6.80%	<0.001
Long acting contraceptive methods	Levoplant	0.0%	0.0%	0.00%	1.3%	1.30%	<0.001
	Jadelle	33.2%	36.6%	37.5%	34.1%	0.90%	0.055
	Implanon	4.6%	6.1%	5.8%	8.7%	4.10%	<0.001
	IUD	0.8%	0.8%	0.8%	0.9%	0.10%	0.475
	Vasectomy	0.01%	0.0%	0.022%	0.00%	−0.01%	0.488
	Tubal ligation	0.02%	0.00%	0.01%	0.0%	−0.02%	0.023
Adults							
Short acting contraceptive methods	Female condom	0.1%	0.1%	0.1%	0.1%	0.00%	0.123
	Male condom	5.9%	2.7%	2.9%	3.6%	−2.30%	<0.001
	Progestin-only pills	2.2%	2.0%	2.2%	1.9%	−0.30%	<0.001
	Combined OCPs	10.1%	10.6%	9.7%	9.5%	−0.60%	<0.001
	Norlevo	0.0%	0.0%	0.00%	0.2%	0.20%	<0.001
	Sayanapress	19.3%	20.9%	23.1%	24.8%	5.50%	<0.001
	Depoprovéra	36.8%	34.7%	31.8%	28.9%	−7.90%	<0.001
Long acting contraceptive methods	Levoplant	0.0%	0.0%	0.00%	0.8%	0.80%	<0.001
	Jadelle	20.8%	22.5%	23.8%	22.0%	1.20%	0.137
	Implanon	2.5%	3.8%	3.4%	5.2%	2.70%	<0.001
	IUD	2.3%	2.6%	2.9%	3.2%	0.90%	<0.001
	Vasectomy	0.00%	0.00%	0.00%	0.00%	0.00%	0.356
	Tubal ligation	0.00%	0.00%	0.00%	0.00%	0.00%	0.500

Age-adjusted analyses revealed *some* distinct patterns of modern contraceptive method upatke across age groups. For example, *t*he decline in male condom uptake *wa*s more pronounced among adolescents (−3.5 percentage points) compared to adult women (−2.3 percentage points). Uptake of combined OCPs remained stable among adolescents at 6.5% though it declined among adult women (−0.6 percentage points). Further, uptake of provider-administered injectables (Depo-Provera) declined in both age groups, with a slightly larger reduction among adults (−7.9 percentage points) than among adolescents (−6.8 percentage points). Conversely, the uptake of self-administered injectables (Sayanapress) increased in both groups, with a greater increase among adults (+5.5 percentage points) compared with adolescents (+4.2 percentage points). Uptake of IUD increased more markedly among adults (+0.8 percentage points). By contrast, uptake of Implanon, a shorter-duration implant method, increased more among adolescents (+4.7 percentage points) than among adults women (+2.1 percentage points) (Table 4).

### Trends in modern family planning stock-outs following the introduction of the free family planning policy

Regarding availability, most CSPS did not experience prolonged stock-outs exceeding three months for modern contraceptive methods. However, extended stock-outs were concentrated on specific commodities. In 2024, stock-outs were concentrated on implants, with nearly one-third of facilities reporting stock-outs lasting more than three months LevoPlant (32.5%) and one-fifth (19.1%) for implan (*p* < 0.01). Progestin-only pills and female condoms showed a marked upward trend in prolonged stock-outs, increasing respectively from 7.9% in 2021 to 13.5% in 2024, and 5.6% in 2021 to 10.9% in 2024 (*p* < 0.01) (Table 5).

**Table 5:** Proportion of PHCs with more than three months of contraceptive stock-outs by facility characteristics 2021–2024.

Method type	Methods	2021	2022	2023	2024	*P*
National						
Short acting contraceptive methods	Female condom	5.6%	6.7%	9.0%	10.9%	<0.01
	Male condom	3.4%	3.2%	3.2%	3.7%	0.66
	Progestin-only pills	7.9%	10.8%	16.9%	13.5%	<0.01
	Combined OCPs	2.2%	2.1%	10.4%	8.5%	<0.01
	Norlevo	na	na	na	33.2%	na
	Sayanapress	2.8%	3.0%	3.4%	4.0%	0.03
	Depoprovéra	1.3%	2.2%	2.0%	3.1%	0.00
Long acting contraceptive methods	Levoplant	na	na	na	32.5%	na
	Jadelle	0.6%	1.1%	1.1%	1.9%	0.00
	Implanon	17.3%	19.3%	27.5%	19.1%	0.00
	IUD	4.6%	4.8%	4.4%	6.9%	0.00
Security challenged area						
Short acting contraceptive methods	Female condom	7.0%	11.5%	11.7%	15.6%	<0.01
	Male condom	4.2%	4.5%	4.8%	4.8%	0.55
	Progestin-only pills	9.6%	17.3%	18.4%	16.1%	0.00
	Combined OCPs	3.3%	3.9%	9.0%	8.8%	<0.01
	Norlevo	0.0%	0.0%	0.0%	29.8%	na
	Sayanapress	3.6%	4.2%	4.2%	3.9%	0.72
	Depoprovéra	2.5%	4.9%	3.3%	3.3%	0.71
Long acting contraceptive methods	Levoplant	na	0.0%	0.0%	29.1%	na
	Jadelle	1.1%	2.2%	1.9%	2.4%	0.11
	Implanon	17.0%	23.0%	27.0%	22.6%	0.00
	IUD	4.0%	6.9%	5.4%	8.9%	0.00
Stable area						
Short acting contraceptive methods	Female condom	4.7%	3.9%	7.5%	8.5%	<0.01
	Male condom	2.9%	2.5%	2.3%	3.1%	0.81
	Progestin-only pills	6.9%	6.9%	16.1%	12.1%	<0.01
	Combined OCPs	1.5%	1.1%	11.2%	8.3%	<0.01
	Norlevo	0.0%	0.0%	0.0%	34.9%	na
	Sayanapress	2.3%	2.3%	2.9%	4.0%	0.01
	Depoprovéra	0.6%	0.7%	1.3%	3.0%	<0.01
Long acting contraceptive methods	Levoplant	na	0.0%	0.0%	34.3%	na
	Jadelle	0.3%	0.5%	0.7%	1.6%	0.00
	Implanon	17.5%	17.1%	27.8%	17.3%	0.06
	IUD	4.9%	3.6%	3.9%	5.8%	0.26
Rural						
Short acting contraceptive methods	Female condom	5.6%	7.0%	9.2%	11.1%	<0.01
	Male condom	3.1%	3.0%	2.9%	3.6%	0.50
	Progestin-only pills	7.6%	11.1%	18.3%	14.5%	<0.01
	Combined OCPs	2.1%	2.1%	10.5%	8.2%	<0.01
	Norlevo	na	na	na	33.0%	na
	Sayanapress	2.7%	2.9%	3.6%	3.8%	0.05
	Depoprovéra	1.4%	2.4%	2.0%	2.8%	0.02
Long acting contraceptive methods	Levoplant	na	na	na	31.9%	na
	Jadelle	0.4%	1.0%	1.1%	1.9%	0.00
	Implanon	17.3%	20.2%	28.0%	19.3%	0.00
	IUD	5.1%	5.0%	4.4%	7.0%	0.06
Urban						
Short acting contraceptive methods	Female condom	5.5%	5.9%	8.4%	10.4%	0.00
	Male condom	4.3%	3.9%	3.9%	4.1%	0.84
	Progestin-only pills	8.7%	10.0%	12.9%	10.6%	0.15
	Combined OCPs	2.4%	2.1%	10.2%	9.3%	<0.01
	Norlevo	na	na	na	33.7%	na
	Sayanapress	3.0%	3.3%	2.7%	4.4%	0.27
	Depoprovéra	1.2%	1.8%	2.2%	3.9%	0.00
Long acting contraceptive methods	Levoplant	na	na	na	34.2%	na
	Jadelle	1.0%	1.6%	1.2%	1.7%	0.41
	Implanon	17.4%	16.8%	26.1%	18.4%	0.12
	IUD	3.2%	4.3%	4.5%	6.6%	0.01

NA, Not available.

These methods were not provided at the time.

Across the post-policy implementation period from 2021 to 2024, facilities located in security-challenged areas consistently reported higher proportions of prolonged stock-outs compared to those in stable settings. In 2024, extended stock-outs in insecure areas affected 22.6% of facilities for implanon, compared with 17.3% in stable areas, and 16.1% for progestin-only pills, compared with 12.1% in facilities based in stable areas. Female condoms similarly exhibited higher levels of prolonged stock-outs in security challenged districts (*p* < 0.01). Prolonged stock-outs of progestin-only pills and female condoms were higher in facilities located in rural area compared to those in urban area (*p* < 0.01) (Table 5).

## Discussion

In this study, we aimed to evaluate contraceptive uptake before and following the implementation of free family planning services under the Gratuité policy in Burkina Faso. Our findings revealed that the removal of user fees did not translate into increased uptake of contraception across the country. Instead, overall family planning uptake significantly declined from 24.3% in the pre-policy period to 21.3% post-policy, which is contrary to the policy expectation. Our finding helps to properly contextualise findings from a previous study by Tiendrebeogo et al., 2023, which was conducted in selected rural and non-conflict areas of Burkina Faso and reported that the user fee removal for family planning services increased modern contraceptive use among WRA by 13% in the months immediately after implementation and the likelihood of using contraception by 86% (95% CI = 49%, 131%) from the pilot period to a year following the national roll-out of the policy (23). Indeed, while the likelihood for uptake increased from pilot to one year after national rollout comparing pilot to non-pilot districts, in reality uptake did not increase across the country comparing pre-implementation period to after the national rollout. This might have more to do with implementation challenges with the national rollout. Indeed, even the pilot phase across two regions had notable Implementation challenges including issues relating to insufficient communication, shortages of contraceptives and consumables, and delays in reimbursement by the government (17). In a different study relating to the Gratuité scheme implementation for maternal newborn and child health, stakeholders highlighted lack of sufficient time for piloting the scheme to inform implementation (18). Focusing on the years which we have not included in our study, 2019 and 2020, both of which constituted a period of deep challenge in global health more broadly as a result of the COVID-19 pandemic, two other studies which included Burkina Faso, reported that the COVID-19 pandemic did not reduce the need and use of modern contracptive in Burkina Faso (14, 40). Put together, all previous studies focused on the immediate aftermath of the policy which also aligns with the initial period of the COVID-19 pandemic (14, 23, 40). While the policy might have helped to improve or stabilise uptake at the time, our longer-term outlook to assess uptake focusing on a period up to three years after the COVID-19 pandemic suggests that those initial gains have not been sustained over time. When disaggregated by region, we observed non-significant changes in uptake some regions in the country, including Centre Est and Centre Nord. However, we found significant decline in family planning uptake between the pre- and post-policy periods in conflict-affected regions including Boucle du Mouhoun, Sahel, and Nord, which all face relatively higher risk of conflict and terrorism (41). This situation has attendant effects on the health system, compromising its capacity for effective modern contraceptives supply chain and making it difficult for women to access and recieve family planning commodities, even though there is a user fee exemption policy (42).

By client characteristic, we found lower utilisation rates amongst adolescents aged 15–19 years compared with older women aged 20–49 years, which is not entirely unexpected and has been reported in similar LMICs (43). However, this finding does not reflect met need and being that our analysis was limited to public sector uptake, it does not negate the possibility that adolescents might be seeking family planning commodities in the private sector, to bypass the steep social barriers, provider bias, and privacy concerns they have reported in the public sector (44, 45). Focusing on the years since the policy implementation, we found that rates amongst both adolescent aged 15–19 years and adult women aged 20–49 years have been on the decline since 2021, though the rate has been faster amongst adult women. The reason for the faster decline amongst adult women is not particularly clear. However, issues relating to limited women's decision-making power in matters of health, particularly with regard to use of family planning, have been reported by adult women, even after the free family planning was added to the Gratuité package (46). Another potential explanation could related to some lingering issues relating to erosion of trust as a result of the several preventive measures implemented during the COVID-19 pandemic—issues that are even more amplified amongst older populations (47). Separately, we found higher utilisation rates amongst women who sought care in facilities based in rural compared to urban areas and a sharp decline in utilisation rates amongst women who sought care in facilities based in rural areas from 2021 to 2024. These patterns are a complete reversal to trends before the policy as shown in a 2015 study which showed lower utilisation rates amongst women in rural compared to urban areas and increasing trends in both rural and urban Burkina Faso between 1998 and 2010 (48). We posit that this sharp rate of decline might also have some link to the lingering issues relating to erosion of trust following the COVID-19 pandemic, which was worse amongst rural populations (47).

While the overall modern contraceptive utilisation has declined over time and since the implementation of the policy, there has been a shift in the contraceptive method used towards long-term methods, though short-acting modern contraceptive methods remained the predominant form of modern contraceptives being used. Specifically, while the proportion of WRA who received short-acting methods declined from 57.3% in 2021 to 54.2% in 2024, long-acting methods uptake increased from 42.7% to 45.8%. The higher rate of short-acting modern ontraceptive methods across several countries in Africa including Burkina Faso has been reported in the literature (35). Indeed short-acting modern contraceptive methods such as condoms tend to be less restricted compared to the more effective long-acting ones with providers known to actively discourage or refuse to prescribe some long acting modern contraceptives to adolescent girls (49). In any case, our observation of the gradual shift towards long-acting modern contraceptive methods, such as implants and IUDs, over short-acting methods like pills and injections aligns with shifting patterns across Africa that have been highlighted in the literature already (50). The shift to long acting methods was also noted in 2020 during the pandemic in a Burkinabé study based on the Performance Monitoring for Action database (51). Subgroup analysis in our study indicated that this transition towards long-acting methods was driven primarily by rural areas, while method uptake patterns remained relatively stable in urban areas. In contrast, the shift was observed consistently across both stable and security-challenged areas. A plausible explanation for the shift in method mix is the benefits associated with long-acting methods over short-acting methods. Evidence suggests that more women are now choosing long-acting methods because this type of modern contraceptive provides longer protection, better child spacing, and greater effectiveness compared to short-acting methods. It is also associated with convenience, and low maintenance (52, 53). Women are also rational beings who would want to maximise profit and minimise risk. Given that there is a high stockout of contraceptive methods in Burkina Faso, women may want to reduce the risk of not getting accessing to a preferred short-acting method, hence, resorting to long-acting methods as evidenced in this study. It is also worth noting that the consistent modest increase in the uptake of Sayanapress indicates that women are becoming more adaptive to family planning commodities and gradually accepting self-care interventions. The increase in Sayana Press uptake may reflect the specific policy promotion that the commodity has been accorded under the Ministry of Health's 2021–2025 Family Planning Strategic Plan (20), which coincided with the introduction of the free family planning as part of the Gratuité package. This is of policy significance as it could mitigate provider shortages and supply chain disruptions that have, over the years, been a bane in advancing modern contraceptive utilisation in Burkina Faso.

Evidence from our study highlights persistent contraceptive stock-outs over the years. Nearly one-third of facilities reported stock-outs exceeding three months for some methods, with implant disruptions peaking at over one-quarter of facilities nationally. Our findings align with Muhoza et al., 2021, whose study found implants to be the most stocked-out contraceptive method in Burkina Faso (35). Our findings also revealed that the stability of the area played a pivotal role. Specifically, facilities located in security-challenged districts reported consistent higher levels of stock-outs compared to facilities located in stable districts. This corroborates earlier studies that have established that in conflict zones or security-challenged contexts, healthcare infrastructure is destructed, healthcare workers are displaced, and access to SRH services (including family planning commodities) is compromised (54). Another key reflection from our findings emerges when we link the observed stockout with the decline in overall family planning uptake in the post-policy period. While it might have been expected that the policy will drive uptake and potentially tip facilities into stock out, our findings suggest that the more likely scenario is that the persistent supply chain issues in the country (35) are more likely the reason for the observed stock-out and not any increased uptake.

### Strengths and limitations

The result from this study provide the first nationwide assessment of the Gratuité policy's effects on both modern family planning adoption and continuation, with a specific focus on equity, adolescents and youth, and conflict-affected settings. Also, the utilisation of large nationally representative datasets covering multiple years provides a robust assessment of the temporal trends and changes. It must also be noted that the use of interrupted time series analysis enhanced causal inference concerning policy timing, whereas district-level modelling captured contextual heterogeneity.

Despite these strengths, there are pertinent limitations to be considered in interpreting the findings. First, we collected data only from public health facilities as per the available datasets. However, this means we were not able to describe modern contraceptivesuptake of distributed through private pharmacies, local NGOs, or community health workers. However, with four of five users of modern contraceptives in Burkina Faso seeking care from within the public sector (30), it is unlikely that or results will be massively affected with private sector utilisation added. Second, our exclusion of 2019 and 2020, though for methodologically sound reasons, precluded us from evaluating the immediate transition effects of the policy. Third, our exclusion of some health facilities that could not be matched with population and service utilisation datasets due to inconsistencies in facility naming across data sources (about 8% of all public health facilities) might have biased our results. However, we believe its impact on study findings is likely limited, as both the population denominators and corresponding service utilisation data for the health facilities were excluded. Nevertheless, this exclusion may introduce some bias in the interpretation of trends, particularly if excluded facilities differ systematically in reporting completeness or service delivery patterns. Fourth, facility catchment WRA were reconstructed using census projections from the national statistics institute and mapped to facility catchment areas. These estimates do not explicitly account for internally displaced populations, which may reduce accuracy in conflict-affected areas. Finally, while we could map specific family planning commodities being used, the available data did not allow us to distinguish the reason for uptake or otherwise as to whether it is related to supply- or demand-side factors.

### Implications for future research, policy and public health practice

Future studies should consider evaluating other aspects of family planning utilisation, including method continuation, discontinuation and method switching. This will make the evidence comprehensive in informing policy action. In addition, qualitative research to help in properly unpacking the reasons underpinning the observed trends and patterns will be a nsensible next research building on this research. There is certainly also a case for comparative analyses across West African conflict and non-conflict countries implementing similar exemption schemes would provide valuable lessons to inform best practices across the sub-region.

In the context of the user fee exemption policy for family planning, women should be entitled to free services, however, stockout of family planning commodities may be contributing to the decline in modern contraceptive use, especially in the fragile and conflict-affected districts of Burkina Faso. Any action to reverse the current trend in modern contraceptive utilisation trend will require confronting the prolonged stockout situation head-on. Policies that targeted family planning coverage focusing on the basic supply of contraceptives in Brazil, Ecuador, Egypt, Ethiopia, and Rwanda have been shown to be successful (55). To address the issue, the Burkinabé government must prioritise strengthening procurement forecasting, buffer stock mechanisms, and decentralised distribution systems. The service delivery points can establish a minimum stock threshold at which point restocking must be done to ensure a continuous and uninterrupted supply of family planning commodities. Also, it is imperative to provide districts with a persistent decline in modern contraceptive use, especially those that are security-challenged and rural, with a differentiated support system. This differentiated support may take the form of emergency supply corridors in insecurity-affected districts, mobile outreach services, and strengthened supervisory mechanisms. It can also manifest through ample resource allocation to areas with evidenced of consistent low modern contraceptive utilisation. The evidence of increased uptake of Sayanapress can be leveraged to scale up access and availability of self-administered methods.

## Conclusion

Based on the findings, we conclude that the free family planning in Burkina Faso has not resulted in a concomitant improvement in overall modern contraceptive uptake and in certain sub-populations across the country. It was rather characterised by a consistent decline in modern contraceptive utilisation, uneven facility readiness, chronic stock-outs, and insecurity-driven supply disruptions. However, self-administered interventions like Sayanapress and long-acting methods have seen a relative increase over time. The findings provide direct evidence that improving the demand-side of family planning without commensurate efforts to enhance the supply-side may hinder the policy from achieving its core mandate of improving modern contraceptive utilisation. For this reason, the Burkinabè government and family planning stakeholders must accelerate efforts to address prolonged stockouts of family planning commodities, especially in security-challenged districts of the country.

## Data Availability

The data analyzed in this study is subject to the following licenses/restrictions: The data supporting this study's findings are available from the Ministry of Health's District Health Information System 2 (DHIS2). Access to these datasets was granted to the authors for the purpose of this study. Requests to access these datasets should be directed to Joel Kiendrebeogo, jkiendre@gmail.com.
